# Development and validation of a nomogram for predicting localization-related complications after CT-guided soft hook-wire placement for solitary pulmonary nodules

**DOI:** 10.3389/fonc.2026.1792100

**Published:** 2026-03-06

**Authors:** Yangfan Zhang, Yining Xu, Jiangnan Dong, Yifeng Zheng

**Affiliations:** 1 Department of Radiology, Huzhou Central Hospital, Fifth School of Clinical Medicine of Zhejiang Chinese Medical University, Huzhou, Zhejiang, China; 2 Department of Radiology, Affiliated Huzhou Hospital, Zhejiang University School of Medicine, Huzhou, Zhejiang, China; 3 Department of Radiology, Huzhou Central Hospital, Affiliated Central Hospital Huzhou University, Huzhou, Zhejiang, China

**Keywords:** complications, CT-guided, nomogram, pulmonary nodule, soft hook-wire

## Abstract

**Background:**

Although the soft hook-wire has been designed to be less traumatic than rigid devices, localization-related complications are still at risk to perioperative safety. This study aimed to find out independent risk factors for these complications in solitary pulmonary nodules patients (SPNs) and create a new nomogram for exact risk stratification.

**Methods:**

Retrospectively analyzed 244 consecutive patients who underwent CT-guided soft hook-wire localization and subsequently had video-assisted thoracoscopic surgery for SPNs in our department from January 2025 to November 2025. Univariate and multivariate logistic regression analysis was done to find out independent predictors for complications. According to these factors, we developed and evaluated a prognosis nomogram.

**Results:**

Technical success rate of localization was 100%. Post-procedural complications occurred in 71 (29.1%) patients. Complications were restricted to pneumothorax and pulmonary hemorrhage. After multivariate analysis, it was found that the presence of emphysema (OR: 15.45, *P* < 0.001), nodule location in the right upper lobe (OR: 6.08, *P* < 0.001), and lower platelet count (OR: 0.99, *P* = 0.012) were all independent risk factors. The well-calibrated model achieved a C-index of 0.80 (95% CI: 0.74–0.86) and demonstrated net clinical benefits across threshold probabilities of 0–0.85.

**Conclusion:**

The prediction nomogram developed in this study exhibits good accuracy in predicting complications following CT-guided soft hook-wire localization of SPNs and provides an objective tool for clinical staff to assess preoperative risk.

## Introduction

The widespread use of low dose computed tomography (LDCT) has substantially increased the detection of pulmonary nodules, particularly ground-glass opacities (1, 2). Although video-assisted thoracoscopic surgery (VATS) remains the standard of care, intraoperative identification of small, deep-seated, or non-solid nodules is challenging due to limited visual and tactile feedback, making preoperative localization often necessary (3–6).

Different CT guided localization methods have been developed because of it, such as methylene blue dye, microcoils, hook-wire system etc. (7–9). Among which the hook-wire technique is considered the most reliable with the highest success rate. However, rigid hook-wires (RHW) also have many shortcomings. The stiffness of the metal wire would cause discomfort to the patient, pleuritic pain, and, more importantly, dislodgement or laceration of the lung parenchyma during patient transport or breathing (10). To avoid these shortcomings, a new soft hook-wire (SHW) has been developed. Unlike the old fixed device, SHW is a flexible suture with a four-hook anchor. This design aims to reduce tension on the pleural surface, thereby improving patient tolerance and potentially reducing mechanical trauma to the lung tissue (5, 10). Recent studies confirm SHW’s clinical superiority as demonstrated by Wang et al., who reported lower rates of pneumothorax (25.8% vs. 28.4%), hemorrhage (13.6% vs. 14.9%), and dislodgement (0% vs. 7.46%) than RHW (10).

Design improvements notwithstanding, pneumothorax and pulmonary hemorrhage continue to be complications. These adverse events affect perioperative safety, necessitate urgent interventions such as thoracic drainage, and may lead to a delay or cancellation of surgery (11, 12). While most current literature compares RHW to other materials, there is little literature researching individual risk factors for SHW (13). Besides that, existing prediction models based on RHW may not be suitable for this special mechanical property of SHW.

Therefore, building a robust predictive tool for SHW localization is necessary. Nomograms are accepted as effective for quantifying individual risk (14). This study aims to develop and validate a prediction model for complications based on clinical and radiological features in solitary pulmonary nodules patients (SPNs) undergoing CT-guided SHW localization.

## Methods

### Patients

This retrospective study was approved by the Ethics Committee of the HuZhou Central Hospital (Approval No.202509004-01). The requirement for informed consent for data analysis was waived due to the retrospective nature of the study, though all patients provided written informed consent prior to the invasive localization procedure.

We reviewed the medical records of patients with pulmonary nodules admitted to our hospital between January 2025 and November 2025. A total of 244 consecutive patients with SPNs who underwent preoperative CT-guided soft hook-wire localization followed by VATS were enrolled. The indication for surgical resection was determined by experienced thoracic surgeons, while the feasibility of the percutaneous puncture path was assessed by interventional radiologists.

**Figure 1:**
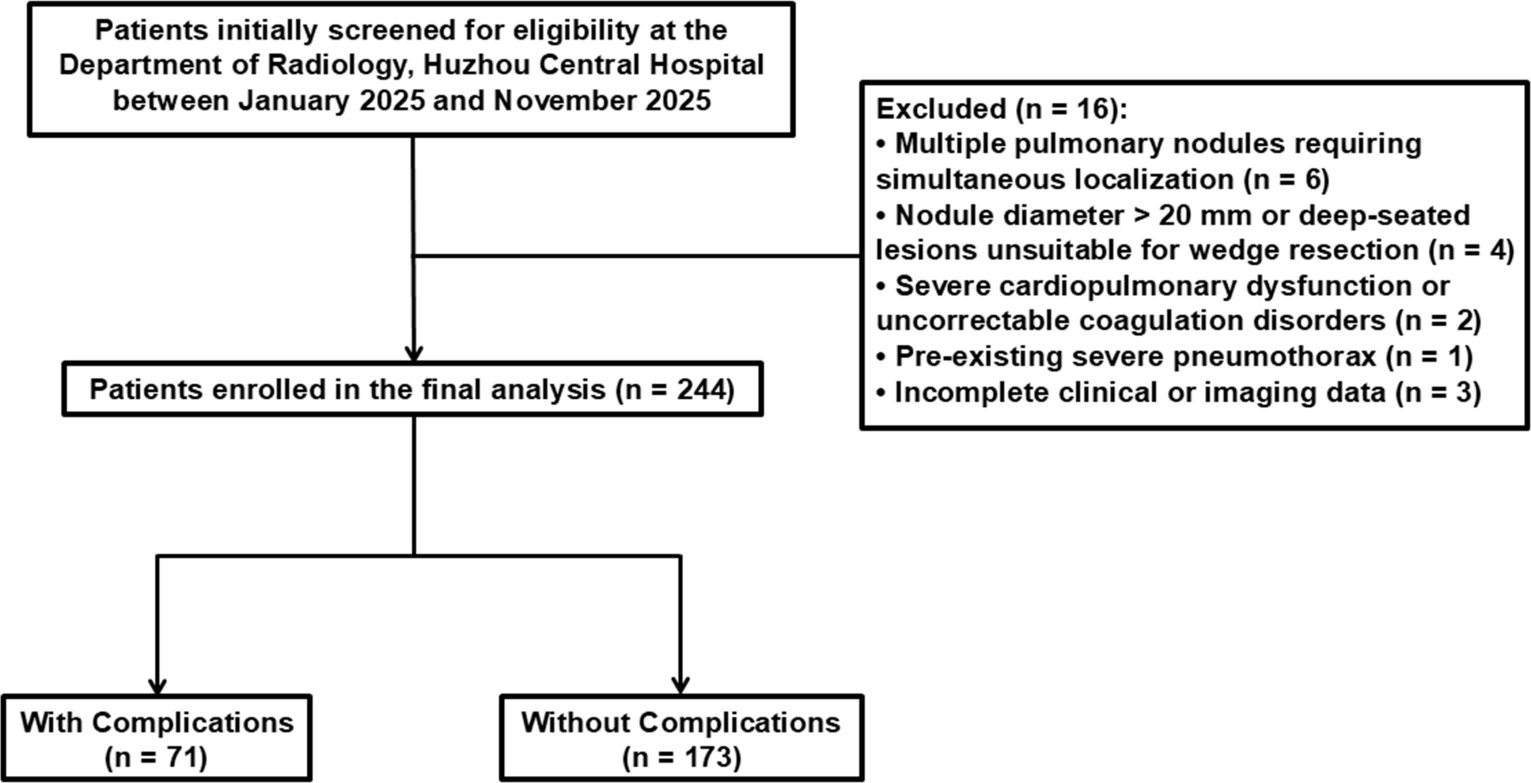
Flowchart of the study population.

The flowchart of patient selection is shown in (**Figure 1**). The inclusion criteria were as follows: (I) age between 18 and 80 years; (II) nodules exhibiting radiological signs of malignancy on CT images; and (III) availability of a safe percutaneous puncture trajectory without obstruction by skeletal structures or major blood vessels. The exclusion criteria were as follows: (I) nodule diameter > 20 mm or deep-seated lesions requiring direct segmentectomy or lobectomy; (II) presence of multiple pulmonary nodules requiring localization; (III) severe cardiopulmonary dysfunction or uncorrectable coagulation disorders; (IV) pre-existing severe pneumothorax, massive pleural effusion, or extensive pleural adhesions interfering with the procedure; and (V) incomplete clinical or imaging data.

Clinical and imaging data were retrospectively retrieved from the Hospital Information System and Picture Archiving and Communication System. Baseline and laboratory variables included age, gender, smoking status, history of malignancy, coagulation profile (platelet count, prothrombin time), and tumor markers (CEA, CA15-3, CA72-4, CA12-5, SCC-Ag). Radiological and procedural variables were observed for emphysema, nodule characteristics (consistency, location, diameter, CT value, spiculation, lobulation, vacuole sign), anatomical parameters (distance to the pleura, chest wall thickness), needle insertion depth, needle entry verticality, and procedure duration.

### Definition of complications

In this study, localization-related complications were assessed based on the confirmatory CT scan performed immediately after SHW placement. Complications included: (1) pneumothorax, which was identified as the presence of gas within the pleural space; (2) hemorrhage, operationally defined as the emergence of new needle-track consolidation or focal ground-glass opacities (GGO) along the puncture trajectory; and (3) other monitored events, such as hemothorax (pleural blood accumulation), wire dislodgement (displacement of the anchor) and wire residue (retained fragments in the lung parenchyma).

### Localization procedure

**Figure 2:**
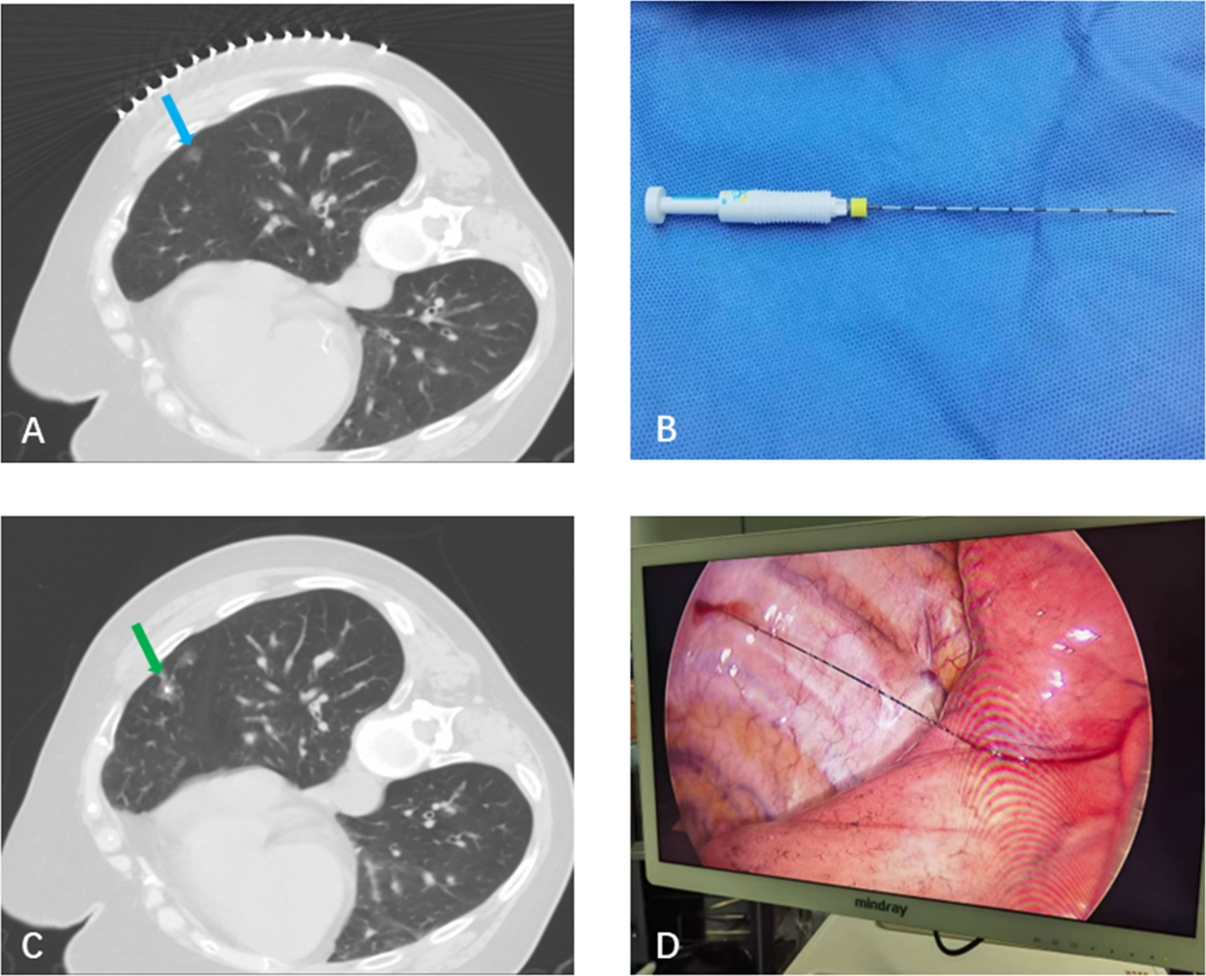
CT-guided localization and VATS resection in an 83-year-old male. **(A)** Initial CT scan showing a ground-glass nodule (blue arrow) in the right middle lobe. **(B)** The disposable soft hook-wire localization needle (Model FLS-20-100). **(C)** Confirmatory CT image to verify the anchor tip position; the green arrow indicates needle track hemorrhage. **(D)** Intraoperative view showing the hookwire located in the right middle lobe.

The patient’s position, such as supine, prone or lateral, is determined by the anatomical location of the nodule. A Philips Brilliance large-bore CT scanner used for the initial scan, with a slice thickness of 3 mm (**Figure 2A**), in order to make better preoperative planning. The optimal puncture path needs to be determined by the operator by assessing the course of adjacent vessels and bone structures. A disposable pulmonary nodule localization needle (Model FLS-20-100; Nanjing Kangyou Medical Technology Co., Ltd.) will be inserted into the lung parenchyma adjacent to the target after completing standard aseptic preparation and local anesthesia (**Figure 2B**). Confirmatory CT imaging will then be generated to verify the position of the anchor tip, which is within 10 mm of the nodule, ruling out complications such as pneumothorax or bleeding (**Figure 2C**) after the anchor being deployed and the cannula being removed.

### VATS resection

All patients were transferred to the operating room for VATS resection within one hour after the localization procedure was completed. The surgical resection was performed under general anesthesia with double-lumen endotracheal intubation for one-lung ventilation. The patient’s position should be lateral decubitus position with the unaffected side down. During the operation, the tail end of the positioning suture originally located inside the chest wall would be pulled back into the thoracic cavity, thus exposing the target lesion (**Figure 2D**). Guided by the localization marker, wedge resection or anatomical segmentectomy was executed, strictly preserving a surgical margin exceeding 2 cm from the nodule periphery.

### Statistical analysis

The statistical analysis was carried out using SPSS v.26.0 and R v.4.2.2. For continuous variables, the mean ± standard deviation or the median (interquartile range, IQR) were calculated and compared using Student’s t-test or Mann-Whitney U test according to normality. Frequencies for categorical variables were calculated and analyzed using Chi-squared or Fisher’s exact test. We used univariate logistic regression to select risk factors that were associated with complications. Variables with a *P* < 0.05 were subsequently entered into a multivariate binary logistic regression analysis using a backward stepwise selection approach. Prior to the multivariate analysis, collinearity among candidate predictor variables was assessed using the Variance Inflation Factor (VIF). A VIF value < 5 was considered indicative of the absence of significant multicollinearity. The model’s performance was internally validated using 1,000 bootstrap re-samples to calculate the optimism-corrected C-index and generate calibration curves. Decision Curve Analysis (DCA) was also performed to evaluate clinical utility. Two-sided p-values less than 0.05 were deemed statistically significant for all analyses.

## Results

### Population

A total of 260 patients were initially screened for eligibility in the department of radiology at Huzhou Central Hospital during the period of January 2025 to November 2025. After careful screening, 16 cases were excluded due to the established criteria, including 6 cases of multiple pulmonary nodules that needed to be located simultaneously, 4 cases of nodules > 20mm or nodules that were too deep and unsuitable for wedge resection, 2 cases of severe cardiopulmonary dysfunction or uncorrectable coagulation disorder, 1 case with severe pneumothorax before surgery, and 3 cases excluded because of incomplete clinical and imaging data. Therefore, a total of 244 cases were enrolled in the final analysis, including 181 females (74.2%) and 63 males (25.8%). The cohort was stratified into two groups based on post-procedural outcomes. In the non-complication group, there were a total of 173 patients (70.9%) with an average age of 55.1 years, including 133 females and 40males. In the complication group, there were 71 patients (29.1%) with an average age of 58.4 years, including 48 females and 23 males.

### Localization outcomes and complications

The success rate of CT-guided soft hook-wire localization was 100% (244/244). All the targeted nodules were accurately marked and successfully resected via subsequent VATS. While the localization-related problems reported by others include a plethora of complications such as pneumothorax, pulmonary hemorrhage, hemoptysis, wire dislodgement, air embolism, and wire residue (6), those noted in the current work were confined to pneumothorax and hemorrhage only. A total of 71 patients (29.1%) experienced post-procedural complications. Specifically, the most common was pneumothorax (54 cases) and pulmonary hemorrhage was noted in 19 cases (2 patients experiencing both). Most pneumothorax cases were minor (lung collapse <30%), with only one patient (1.8%) requiring chest tube drainage while the rest were managed conservatively. One case of intercostal artery injury occurred but resolved spontaneously without intervention.

### Baseline characteristics and factor analyses

To determine the independent predictors for localization-related complications, univariate and multivariate logistic regression analysis was performed with the baseline characteristics as shown in **Table 1**. Multivariate analysis identified three independent risk factors (**Table 2**). Regarding specific predictors, the presence of emphysema emerged as the strongest risk factor in terms of odds ratio (OR, 15.45; 95% CI, 3.16–75.62; *P* < 0.001), followed by nodule location in the right upper lobe (OR, 6.08; 95% CI, 2.42–15.29; *P* < 0.001). Also, the platelet count was negatively correlated with the risk of complications (OR, 0.99; 95% CI, 0.982–0.998; *P* = 0.012), while the other covariates did not show statistical significance in the multivariate model (*P* > 0.05).

**Table 1:** Baseline characteristics of the study population.

Variables	Total (n = 244)	Non-complication group (n = 173)	Complication group (n = 71)	Statistic	*P*
Age (years)	56.07 ± 13.12	55.10 ± 13.26	58.44 ± 12.57	t=-1.81	0.071
Sex				χ^2^ = 2.26	0.133
Female	181 (74.18)	133 (76.88)	48 (67.61)		
Male	63 (25.82)	40 (23.12)	23 (32.39)		
Smoking history				χ^2^ = 2.77	0.096
No	207 (84.84)	151 (87.28)	56 (78.87)		
Yes	37 (15.16)	22 (12.72)	15 (21.13)		
History of malignancy				χ^2^ = 0.48	0.488
No	207 (84.84)	145 (83.82)	62 (87.32)		
Yes	37 (15.16)	28 (16.18)	9 (12.68)		
Emphysema				χ^2^ = 39.17	<.001
No	224 (91.80)	171 (98.84)	53 (74.65)		
Yes	20 (8.20)	2 (1.16)	18 (25.35)		
Prothrombin time (s)	11.08 ± 0.69	11.12 ± 0.71	10.99 ± 0.64	t=1.41	0.16
Platelet count (109/L)	208.61 ± 58.87	217.50 ± 61.44	186.94 ± 45.67	t=4.27	<0.001
Tumor markers					
CEA (ng/mL)	2.08(1.47,2.70)	2.06(1.37,2.64)	2.15(1.61,2.79)	Z=−1.30	0.192
CA15-3 (U/mL)	9.20(6.50,11.83)	9.50(6.70,11.90)	8.40(6.10,11.70)	Z=−0.95	0.344
CA72-4 (U/mL)	2.94(2.21,4.77)	2.96(2.18,4.77)	2.93(2.30,4.27)	Z=−0.42	0.675
CA12-5 (U/mL)	13.20(9.40,16.80)	13.60(9.30,17.30)	12.00(10.00,15.30)	Z=−0.85	0.397
SCC (ng/mL)	0.78(0.61,0.99)	0.76(0.63,0.93)	0.84(0.57,1.21)	Z=−1.21	0.226
Nodule characteristics					
Nodule type				χ^2^ = 4.12	0.128
Solid	46(18.9)	27(15.6)	19(26.8)		
Ground-glass opacity (GGO)	170(69.7)	125(72.3)	45(63.4)		
Mixed GGO	28(11.5)	21(12.1)	7(9.9)		
Mean diameter (mm)	7.00(5.00,9.00)	7.00(5.00,9.00)	8.00(5.50,9.00)	Z=−0.63	0.531
Distance to pleura (mm)	8.80(3.50,16.12)	8.30(3.20,15.60)	9.80(4.65,18.40)	Z=−1.47	0.14
Mean CT value (HU)	−443.6 ± 233.2	−456.9 ± 216.4	−411.1 ± 268.8	t=−1.28	0.204
Vacuole sign				χ^2^ = 1.92	0.166
Absent	204(83.6)	141(81.5)	63(88.7)		
Present	40(16.4)	32(18.5)	8(11.3)		
Lobulation				χ^2^ = 0.26	0.607
Absent	217(88.9)	155(89.6)	62(87.3)		
Present	27(11.1)	18(10.4)	9(12.7)		
Spiculation				χ^2^ = 0.23	0.634
Absent	233(95.5)	164(94.8)	69(97.2)		
Present	11(4.5)	9(5.2)	2(2.8)		
Location				χ^2^ = 35.16	<0.001
Left upper lobe	62(25.4)	53(30.6)	9(12.7)		
Left lower lobe	37(15.2)	30(17.3)	7(9.9)		
Right upper lobe	75(30.7)	34(19.7)	41(57.7)		
Right middle lobe	20(8.2)	15(8.7)	5(7.0)		
Right lower lobe	50(20.5)	41(23.7)	9(12.7)		
Procedural parameters					
Needle path length (mm)	13.61 ± 3.07	13.49 ± 2.87	13.90 ± 3.52	t=−0.96	0.338
Chest wall thickness (mm)	39.92 ± 12.17	40.32 ± 12.30	38.95 ± 11.88	t=0.80	0.423
Puncture duration (min)	10.0(8.0,13.3)	10.0(8.0,13.0)	11.0(8.0,15.5)	Z=−1.25	0.21
Vertical to pleura				χ^2^ = 0.04	0.843
No	111(45.5)	78(45.1)	33(46.5)		
Yes	133(54.5)	95(54.9)	38(53.5)		

Data are presented as the mean ± standard deviation for normally distributed continuous variables, the median (interquartile range [Q1, Q3]) for non-normally distributed variables, or number (percentage) for categorical variables. t, t-test; Z, Mann-Whitney test; χ^2^, Chi-square test.

**Table 2:** Multivariate logistic regression analysis of independent risk factors for complications.

Variables	β	P	OR (95%CI)
Emphysema	2.74	<.001	15.45 (3.16 ~ 75.62)
Right upper lobe†	1.80	<.001	6.08 (2.42 ~ 15.29)
Platelet count	-0.01	0.012	0.99 (0.982–0.998)

OR, Odds Ratio; CI, Confidence Interval; ^†^Location was analyzed using dummy variable encoding, where the Left Upper Lobe served as the reference category.

### Construction of the nomogram

**Figure 3:**
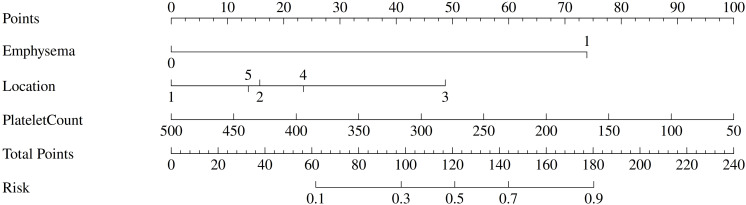
Nomogram for predicting the probability of complications after CT-guided soft hook-wire localization. The variable “Location” is categorized as follows: 1, Left Upper Lobe; 2, Left Lower Lobe; 3, Right Upper Lobe; 4, Right Middle Lobe; 5, Right Lower Lobe.

The predictive nomogram (**Figure 3**) was constructed by utilizing independent risk factors after being validated by multivariate analysis (*P* < 0.05). Under this framework, each predictor was assigned a score on the upper axis, with the cumulative score then being mapped to the lower axis to quantify the specific probability of localization-related complications. Moreover, platelet count is the most important in the model prediction, being negatively correlated with the total score. In other words, a lower platelet level means a higher risk score. Additionally, complications are more likely to occur if the total score is increased due to the presence of emphysema and lesions in the right upper lobe.

### Validation and clinical utility

**Figure 4:**
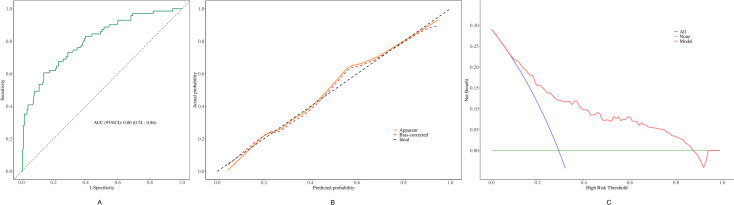
Evaluation of the nomogram model. **(A)** Receiver operating characteristic curve. **(B)** Calibration curve. **(C)** Decision curve analysis.

Nomogram performance was rigorously verified through internal validation using 1,000 bootstrap re-samples. To be more specific, the discrimination ability was determined by ROC curve (**Figure 4A**), with the optimism-corrected C-index reaching 0.80 (95%CI: 0.74 - 0.86), indicating its strong discriminatory ability and good predictive performance. The calibration curve in **Figure 4B** shows that the bias-corrected curve closely aligned with the ideal reference line, which means that the predicted probabilities and the observed outcome have good concordance. To further examine the clinical application prospects, DCA was carried out (**Figure 4C**). It showed that using this nomogram to predict complications would provide a large net clinical benefit for a very wide range of threshold probabilities (0 to 0.85).

## Discussion

Within the paradigm of precision thoracic surgery, preoperative localization serves as the critical pivot connecting radiological identification to VATS resection. In this retrospective analysis of 244 solitary pulmonary nodule cases, the soft hook-wire system yielded a 100% technical success rate, validating its instrumental reliability. Nevertheless, 29.1% of the patients were found to have complications, including pneumothorax and bleeding. This study establishes the first prognostic nomogram specifically designed for soft-hook wire localization in patients with SPNs, integrating factors such as pulmonary emphysema status, nodule location, and platelet count, demonstrating good predictive discrimination (C-index: 0.80) and calibration. Clinicians can better implement risk stratification management plans through this quantitative tool, which can also help in preventive measures and perioperative optimization decisions.

The analysis indicates that emphysema is a key independent predictor of surgical adverse reactions, being consistent with previous literature regarding transthoracic biopsy and hook wire localization (15–17). Pathologically, the alveolar walls are damaged and the alveolar cavities are permanently enlarged due to emphysema, thus damaging the elasticity and structural integrity of the lung parenchyma. The needle tract is difficult to spontaneously close after the puncture needle is withdrawn as the elastic recoil force being weakened when such tissues are penetrated (18, 19). Therefore, pneumothorax will be caused because the unsealed puncture tract will become a channel allowing air to enter the pleural cavity from the alveoli. Moreover, the risk of bleeding will be increased because the damaged pulmonary microvessels in these areas are more susceptible to shear force during the puncture operation or respiratory movement. Therefore, it is recommended to adopt a detailed surgical plan to avoid emphysema in the puncture path.

One notable finding is the independent correlation between right upper lobe localization and an increased probability of complications. Although previous studies have indicated that lower lobe punctures are riskier due to greater diaphragmatic motion (20–22), our research results contradict this and demonstrate the influence of regional physiological heterogeneity. We speculate that the susceptibility of the right upper lobe is influenced by the vertical direction of pleural pressure gradient. Physiologically, the pulmonary apical alveoli will experience inherent over-expansion because the negative pressure of the pleura at the pulmonary apex is significantly higher than that at the pulmonary base (23). Therefore, the pleura is prone to separation and air leakage after puncture because of the inherent over-expansion of the pulmonary apex and the reduction of lung tissue support, regardless of whether the supine position or prone position is adopted. Additionally, this risk may be further exacerbated if the operation of the puncture needle is restricted because of the narrowness of the upper thoracic rib spaces, with the shear stress at the pleural interface also being increased as a result. However, these physiological explanations are speculative and warrant further investigation in prospective studies with larger cohorts to confirm the underlying mechanisms.

The negative correlation was revealed by our analysis between platelet count and the frequency of adverse reactions. Moreover, low normal values were also considered as potential risk factors. The linear risk continuum was supported by our data and is related to platelet abundance, which is different from definite severe thrombocytopenia, being a well-known contraindication. Furthermore, platelets are also important for pulmonary parenchymal micro-defects caused by rapid mechanical closed needle puncture, apart from primary hemostasis (24). In this surgical situation, bleeding events are usually manifested as needle-tract consolidation or GGO around the lesion on imaging (25). A strong thrombus formation response is helpful for immediate vascular occlusion. Most importantly, stable intrapulmonary parenchymal blood clots can also serve as an airway barrier, thereby reducing the incidence of pneumothorax by preventing the escape of alveolar gas. These research results emphasize the necessity of meticulous preoperative hematological assessment. For patients with platelet counts at the critical value, we recommend the use of finer instruments and more rigorous movement restrictions to ensure better occlusion of the puncture tract.

It is worth noting that multivariate analysis revealed that there was no statistically significant correlation between the incidence of complications and surgical indicators, such as puncture depth, puncture angle, thoracic wall thickness, or operation duration, indicating the mechanical advantage of the soft hook wire system. To be more specific, the safety of the surgery is less dependent on the operator’s differences, being completely different from the traditional rigid structure. This is because the rigid metal components act as fulcrums or lever arms in traditional rigid positioning, so a huge shear torque is generated at the visceral pleura even due to some minor angle deviations or changes in respiratory amplitude. In other words, the safety of the surgery is determined by the operator’s dexterity. On the contrary, the flexible sutures with high lung parenchymal compliance are adopted by the soft system, so the sutures can maintain dynamic synchronization with respiratory movements, meaning no resistance being generated after the surgery. In other words, the “cheese wire” effect of the visceral pleura is alleviated by this mechanical decoupling, not being related to the puncture trajectory or depth. Ultimately, the learning curve of the technology is effectively reduced by the system’s fault tolerance, with the safety performance being robustly maintained despite inevitable procedural differences.

Although we have demonstrated the potentials of our nomogram, it is still subject to some limitations. First, as a retrospective single-center study, inherent selection bias is unavoidable and our results may represent the specific technical practice at our institution. Second, although the sample size of 244 patients was adequate for model building, a large external validation cohort should be established to validate the nomogram. Third, we created a composite endpoint of “complications” by combining pneumothorax and hemorrhage because the rate of hemorrhage alone was quite low; larger data sets in the future might develop separate models for each outcome. Fourth, operator experience was not quantitatively analyzed. Although the soft hook-wire system minimizes technical dependency, the expertise of interventional radiologists may still influence procedural outcomes in complex cases. Finally, cases with multiple nodules were excluded to isolate lesion-specific risk factors. Consequently, the generalizability of this model to patients requiring simultaneous multi-lesion localization—an increasingly common clinical scenario—may be restricted.

## Conclusions

In this study, we successfully developed and validated a novel nomogram for predicting complications following CT-guided soft hook-wire localization of SPNs. The model incorporates three readily accessible independent predictors: the presence of emphysema, nodule location in the right upper lobe, and lower platelet count. This nomogram serves as a practical, easy-to-use tool for interventional radiologists and thoracic surgeons to preoperatively stratify patient risk.

## Data Availability

The raw data supporting the conclusions of this article will be made available by the authors, without undue reservation.

## References

[1] Wang C Shao J He Y Wu J Liu X Yang L . Data-driven risk stratification and precision management of pulmonary nodules detected on chest computed tomography. Nat Med. (2024) 30:3184–95. doi: 10.1038/s41591-024-03211-3, PMID: 39289570 PMC11564084

[2] Yoshida R Yoshizako T Tanaka S Ando S Nakamura M Kishimoto K . CT-guided color marking of impalpable pulmonary nodules prior to video-assisted thoracoscopic surgery. Clin Imaging. (2021) 74:84–8. doi: 10.1016/j.clinimag.2021.01.003, PMID: 33454581

[3] Solli P Spaggiari L . Indications and developments of video-assisted thoracic surgery in the treatment of lung cancer. Oncologist. (2007) 12:1205–14. doi: 10.1634/theoncologist.12-10-1205, PMID: 17962614

[4] Yang SM Hsu HH Chen JS . Recent advances in surgical management of early lung cancer. J Formosan Med Assoc. (2017) 116:917–23. doi: 10.1016/j.jfma.2017.07.009, PMID: 28781098

[5] Li GC Wu ZL Shi YB Wang JY Fu YF . Preoperative computed tomography-guided soft hook-wire localization for multiple pulmonary nodules. Front Oncol. (2025) 15:1501165. doi: 10.3389/fonc.2025.1501165, PMID: 40061898 PMC11886374

[6] Yin C Chen Y Zhang R Chen A Fang H Liu W . Analysis of complication risk factors in preoperative computed tomography-guided hookwire location of pulmonary nodules. Eur J Med Res. (2024) 29:369. doi: 10.1186/s40001-024-01970-w, PMID: 39014473 PMC11253328

[7] Zhang X Miao L Che Y Tian P Li J Peng Q . A comparative study of 4-hook anchor device with methylene blue for preoperative pulmonary nodule localization. Quant Imaging Med Surg. (2025) 15:395–403. doi: 10.21037/qims-24-1535, PMID: 39839047 PMC11744168

[8] Sun X Fu J Ma C Song Z Yang S Jin L . CT-guided microcoil versus hook-wire localization of pulmonary nodule prior to video-assisted thoracoscopic surgery without fluoroscopic guidance. BMC Pulm Med. (2024) 24:492. doi: 10.1186/s12890-024-03306-0, PMID: 39379924 PMC11463161

[9] Hu L Gao J Chen C Zhi X Liu H Hong N . Comparison between the application of microcoil and hookwire for localizing pulmonary nodules. Eur Radiol. (2019) 29:4036–43. doi: 10.1007/s00330-018-5939-4, PMID: 30631924

[10] Wang Y Jing L Liang C Liu J Wang S Wang G . Comparison of the safety and effectiveness of the four-hook needle and hook wire for the preoperative positioning of localization ground glass nodules. J Cardiothorac Surg. (2024) 19:35. doi: 10.1186/s13019-024-02497-1, PMID: 38297385 PMC10829251

[11] Wang Z Liang S Lu X Li X Sun D . Severe cerebral air embolism after CT-guided hook-wire localization – complete recovery and delayed lung resection: A case report. Medicine. (2025) 104:e45710. doi: 10.1097/MD.0000000000045710, PMID: 41261591 PMC12582805

[12] Hanauer M Perentes JY Krueger T Ris HB Bize P Schmidt S . Pre-operative localization of solitary pulmonary nodules with computed tomography-guided hook wire: report of 181 patients. J Cardiothorac Surg. (2016) 11:5. doi: 10.1186/s13019-016-0404-4, PMID: 26772183 PMC4715360

[13] Meng Q Wang J Wang X Sun Q . Preoperative computed tomography-guided localization for pulmonary nodules: a systematic review and meta-analysis of soft hook-wire and coil localization. Quant Imaging Med Surg. (2025) 15:6705–12. doi: 10.21037/qims-2025-56, PMID: 40785912 PMC12332689

[14] Chen S Ou R Wei Q Fu J Zhao B Chen X . Identification of risk factors and development of a predictive nomogram for sarcopenia in Alzheimer’s disease. Alzheimer’s Dementia. (2025) 21:e14503. doi: 10.1002/alz.14503, PMID: 39778036 PMC11848345

[15] Zhao Y Wang X Wang Y Zhu Z . Logistic regression analysis and a risk prediction model of pneumothorax after CT-guided needle biopsy. J Thorac Dis. (2017) 9:4750–7. doi: 10.21037/jtd.2017.09.47, PMID: 29268546 PMC5721042

[16] Wu HL Yan GW Lei LC Du Y Niu XK Peng T . Development and validation of a random forest risk prediction pneumothorax model in percutaneous transthoracic needle biopsy. Med Sci Monit. (2021) 27:e932137. doi: 10.12659/MSM.932137, PMID: 34887374 PMC8669970

[17] Zeng L Liao H Ren F Zhang Y Wang Q Xie M . Pneumothorax induced by computed tomography guided transthoracic needle biopsy: A review for the clinician. IJGM. (2021) 14:1013–22. doi: 10.2147/IJGM.S302434, PMID: 33790630 PMC8001193

[18] Kazerooni EA Lim FT Mikhail A Martinez FJ . Risk of pneumothorax in CT-guided transthoracic needle aspiration biopsy of the lung. Radiology. (1996) 198:371–5. doi: 10.1148/radiology.198.2.8596834, PMID: 8596834

[19] Asai N Kawamura Y Yamazaki I Sogawa K Ohkuni Y O’uchi T . Is emphysema a risk factor for pneumothorax in CT-guided lung biopsy? SpringerPlus. (2013) 2:196. doi: 10.1186/2193-1801-2-196, PMID: 23741641 PMC3664182

[20] Hiraki T Mimura H Gobara H Shibamoto K Inoue D Matsui Y . Incidence of and risk factors for pneumothorax and chest tube placement after CT fluoroscopy–guided percutaneous lung biopsy: retrospective analysis of the procedures conducted over a 9-year period. Am J Roentgenol. (2010) 194:809–14. doi: 10.2214/AJR.09.3224, PMID: 20173164

[21] Chami HA Faraj W Yehia ZA Badour SA Sawan P Rebeiz K . Predictors of pneumothorax after CT-guided transthoracic needle lung biopsy: the role of quantitative CT. Clin Radiol. (2015) 70:1382–7. doi: 10.1016/j.crad.2015.08.003, PMID: 26392317

[22] Huang MD Weng HH Hsu SL Hsu LS Lin WM Chen CW . Accuracy and complications of CT-guided pulmonary core biopsy in small nodules: a single-center experience. Cancer Imaging. (2019) 19:51. doi: 10.1186/s40644-019-0240-6, PMID: 31337425 PMC6651998

[23] Agostoni E . Mechanics of the pleural space. Physiol Rev. (1972) 52:57–128. doi: 10.1152/physrev.1972.52.1.57, PMID: 4550113

[24] Ball K Pokrovskaya I Storrie B . Puncture wound hemostasis and preparation of samples for montaged wide-area electron microscopy analysis. JoVE. (2024) 24:207. doi: 10.3791/66479, PMID: 38856226 PMC11637564

[25] Filippo MD Saba L Silva M Zagaria R Concari G Nizzoli R . CT-guided biopsy of pulmonary nodules: is pulmonary hemorrhage a complication or an advantage? Diagn Interv Radiol. (2014) 20:421–5. doi: 10.5152/dir.2014.14019, PMID: 25163758 PMC4463325

